# Use of a Tissuetunneler guide for an automatic suture device in robot-assisted thoracic surgery

**DOI:** 10.1007/s11748-025-02141-2

**Published:** 2025-05-20

**Authors:** Tomohiko Matsuzaki, Hiroto Onozawa, Atsushi Wada, Naohiro Aruga, Masayuki Iwazaki, Ryota Masuda

**Affiliations:** Department of Thoracic Surgery, Tokai University School of Medicine, 143 Shimokasuya, Isehara, Kanagawa 259-1193 Japan

**Keywords:** Robot-assisted thoracic surgery, Tissuetunneler, SureForm stapler curved tip

## Abstract

The SureForm stapler curved tip is a useful approach for vessels. Unless adequate space is maintained below the vessels, damage to the back of vessels with the curved tip may occur. The recommended methods include passing silk threads or vessel loops under the vessels and applying traction or using a Penrose drain to guide the tip of the stapler during insertion. However, these methods often risk putting tension on the vessels or becoming caught in the surrounding tissues. Additionally, the Penrose drain is soft, causing difficultly in guiding the stapler effectively. Furthermore, after removing the Penrose drain, there is a risk of accidental vascular injury by the tip of the stapler. To improve safety and efficiency, we devised a new method using a Tissuetunneler, which guides the stapler properly, prevents entanglement, and allows vessel transection without removal, reducing surgical time.

## Introduction

The widespread adoption of video-assisted thoracic surgery (VATS) and robot-assisted thoracic surgery (RATS) has been supported by the use of staplers, and pulmonary vessels are now commonly managed with staplers [1]. These fragile vessels are difficult to repair and can lead to fatal outcomes if mishandled, necessitating extreme caution [2, 3]. To date, only straight staplers have been used, but these can pose risks when approaching vessels. Recently, the Endo GIA™ curved tip (Covidien, Norwalk, CT, USA) and Powered ECHELON FLEX 7(Ethicon Endo-Surgery, Inc., Cincinnati, OH, USA) have been developed as safe approaches for vascular handling [4, 5]. The SureForm stapler (Intuitive Inc., Sunnyvale, CA, USA) with a curved tip, similar to the Endo GIA™ curved tip and Powered ECHELON FLEX 7, can perform stapling. Furthermore, the SureForm stapler allows for finer angle adjustments compared to these two staplers. However, because RATS lacks tactile feedback, extreme caution is required when tunneling for vascular handling during surgery compared with VATS. The recommended method involves passing silk threads or vessel loops behind the pulmonary vessels and inserting the stapler while applying traction. Alternatively, methods using a Penrose drain to guide the tip of the endostapler have also been attempted. However, several issues remain. These methods often risk applying tension to the pulmonary vessels or becoming caught in the surrounding tissues. Additionally, the Penrose drain is very soft, which can make guiding the stapler difficult. Furthermore, after removing the Penrose drain, there is a risk of accidental vascular injury caused by the tip of the stapler.

To address these challenges, we describe here a new method using a Tissuetunneler (Fuji Systems, Tokyo, Japan) to guide the stapler more safely and efficiently. This device is reportedly approved for use in Japan (Approval number: 22800BZX00397000).

## Materials and methods

**Fig. 1:**
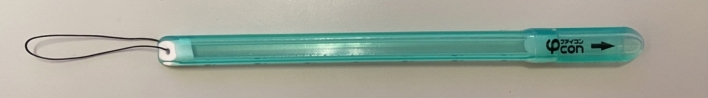
This guide is made of a soft silicone tube with an inner diameter of 6 mm, a thickness of 4 mm, and a length of 8 cm, featuring a smooth surface

**Fig. 2:**
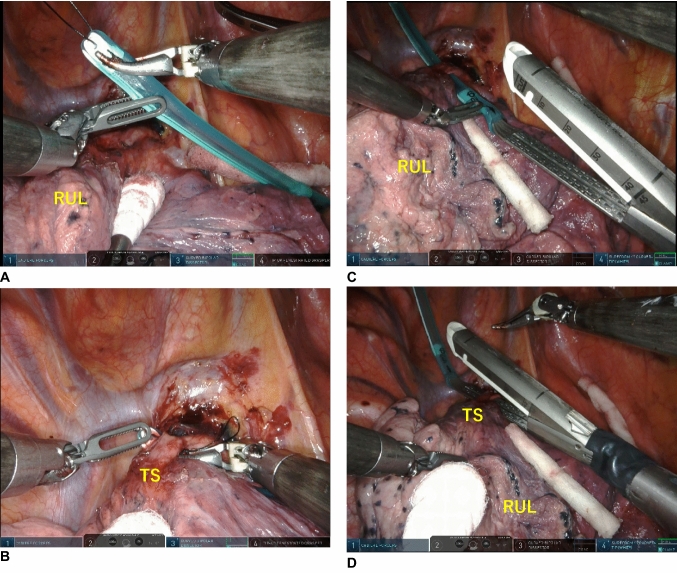
Five ports RATS for right upper lobectomy. **A**, **B** The tip of the Tissuetunneler is grasped using a curved bipolar forceps to perform dissection, followed by tunneling. **C**, **D** The distal end of the anvil of the SureForm stapler curved tip is attached to the caudal end of the Tissuetunneler to guide and complete stapling. RUL, right upper lobe; TS, Truncus superior pulmonary artery

The Tissuetunneler guide is made of a soft silicone tube with an inner diameter of 6 mm, a thickness of 4 mm, and a length of 8 cm, with a smooth surface (Fig. 1). The tip of the Tissuetunneler is grasped using a curved bipolar forceps or Cadiere forceps (Intuitive Inc.) to perform dissection, followed by tunneling (Fig. 2A, B). Subsequently, the distal end of the anvil of the SureForm stapler curved tip is attached to the caudal end of the Tissuetunneler to guide and complete stapling (Fig. 2C, D).

From December 2021 to September 2024, the Tissuetunneler was used in 84 RATS cases for lobectomy. Lobectomies were performed on 31 right upper lobes, 7 right middle lobes, 28 right lower lobes, 5 left upper lobes, and 13 left lower lobes. In all cases, stapler insertion was carried out smoothly (Table 1).

**Table 1:** Details of surgery and postoperative results

Variables	*n* = 84
Laterality (right/left)	(66/18)
Lobectomy	
RUL	31
RML	7
RLL	28
LUL	5
LLL	13
Surgery time (min)	159.9 ± 44.9 (102–327)
Console time (min)	106.3 ± 42.3 (46–277)
Number of harvested lymph nodes	5.3 ± 3.0 (0–14)
Blood loss (mL)	17.9 ± 32.1 (1–202)
Duration of chest tube drainage (day)	2.4 ± 1.1 (1–8)
Postoperative length of stay (day)	3.8 ± 1.1 (2–9)
Conversion of thoracotomy	0
Conversion to VATS	5 (6%)
Morbidity	3 (4%)
Mortality	0

Values are presented as mean ± standard deviation [range], as appropriate

*RUL* right upper lobectomy, *RML* right middle lobectomy, *RLL* right lower lobectomy, *LUL* left upper left upper, *LLL* left lower lobectomy, *VATS* video-assisted thoracic surgery

The study was conducted in accordance with the Declaration of Helsinki (as revised in 2013). The study was approved by the ethics board of Tokai University Hospital (No. 24R126), and informed consent was obtained from all of the patients. The data of the patients were retrospectively evaluated in the present study.

The surgical and postoperative outcomes were assessed using descriptive statistics. Continuous data are presented as the mean ± standard deviation (range) and categorical data are presented as the number and percentage. All collected data were tabulated using Microsoft Excel version16 (Microsoft Store, Tokyo, Japan) and for further analysis.

## Results

No misfiring or accidental injury to vessels or surrounding tissues occurred with the use of the SureForm stapler curved tip. The mean surgery time was 159.9 ± 44.9 min (range 102–327 min), and the mean console time was 106.3 ± 42.3 min (range 46 to 277 min). The mean intraoperative blood loss volume was 17.9 ± 32.1 mL (range 1–202 mL). During vessel management, no vascular injuries occurred with stapling using the Tissuetunneler, and only bleeding from the chest wall due to adhesion dissection was observed.

Conversion to VATS was required in five (6%) cases. That was not due to a technical issue with the Tissuetunneler but rather caused by patient factors such as severe adhesions or difficulties in lung isolation.

There were no complications or perioperative deaths (Table 1).

## Comment

The SureForm stapler curved tip provides easier access to vessels compared with straight staplers. However, if adequate space is not created beneath the vessel, there is a risk of the tip damaging the backside of the vessel. This risk is increased in RATS, where the lack of tactile feedback may lead to excessive force being applied, necessitating extra caution. Therefore, a safer and simpler method for guiding the curved tip is required.

Staplers are typically guided after securing the vessels with silk threads. When we considered that the vascular sheath or surrounding tissues might get caught in the stapler, Penrose drains were used instead of silk threads to secure the vessel [6]. Therefore, Penrose drains have been used as a guide for staplers. However, when suturing near the hilum within the thoracic cavity, the Penrose drain often needs to be removed from the jaws of the stapler. Additionally, because of the extreme softness of Penrose drains, they may not effectively guide the stapler behind the vessel.

However, the 8-cm Tissuetunneler is compact and can be attached to the jaws of the stapler during thoracic operations in RATS. The caudal side of the Tissuetunneler features a semicircular hole where the jaws of the stapler are attached. This design helps prevent the stapler from slipping off. The Tissuetunneler has the appropriate amount of flexibility to guide the stapler’s tip effectively, ensuring that it avoids entangling other tissues and damaging surrounding organs. Additionally, this method allows for the transection of vessels, bronchi, and lung tissue without removing the Tissuetunneler from the stapler. This situation simplifies an intra-thoracic operation and eliminates the risk of damage to other vessels and lung tissue caused by the jaws. Furthermore, the Tissuetunneler attaches only to the tip of the stapler and does not reach the staple area, which provides an advantage over the Penrose drain by preventing the accidental stapling of its plastic component.

For example, the Tissuetunneler serves as an effective and safe guiding tool when stapling delicate vessels such as a narrow ascending A2b in right upper lobectomy or a deeply located A3 in left upper lobectomy. Compared to a Penrose drain, the Tissuetunneler has an optimal level of stiffness, allowing for smooth tunneling without placing excessive tension on the vessel to be stapled. Additionally, it enables visualization of the stapler’s trajectory, making it easier to guide it accurately to the appropriate position, thereby enhancing safety. Furthermore, when passing through the interlobar fissure in fissureless, the Tissuetunneler may contribute to reducing operative time by allowing for minimal dissection and continuous tunneling compared to a Penrose drain.

The thread attached to the tip is designed to make the Tissuetunneler easier to handle and remove from the body.

## Conclusion

Our early results suggest that the Tissuetunneler method is safe, feasible, and provides excellent perioperative outcomes.

## Data Availability

Not applicable.
